# 

**Published:** 1881-03

**Authors:** 


Vol. I. MARCH, 1881. No. 3.

### THE HOMŒOPATHIC PHYSICIAN:

A MONTHLY JOURNAL OF MEDICAL SCIENCE.

"*Magna est veritas et praevalebit.*"

E. J. LEE, M. D.
EDITOR.

*All articles for publication, books for review, &c., must be sent, post-paid, to Editor, 2109 Chestnut Street, Philadelphia; Business communications, to Publishers.*

NEW YORK.
BEDELL & BROTHER, PUBLISHERS,
3rd Avenue and 175th Street.

LONDON.

ALFRED HEATH & CO., SOLE AGENTS FOR GREAT BRITAIN.

114 Ebury Street, Eaton Square.

Yearly Subscription, $2.00, in advance. - - Single Numbers, 25 cents.

Entered at the Post Office at New-York, as Second-Class Mail Matter.

